# The Incidence of *Demodex folliculorum* in the Combination of Allergic Rhinitis and Diabetes Mellitus

**Published:** 2019

**Authors:** Cengiz ARLİ, Muge OZSAN, Eren GURKAN, Ozlem AYCAN KAYA, Sumeyya KOKACYA

**Affiliations:** 1. Department of Otolaryngology, School of Medicine, Mustafa Kemal University, Hatay, Turkey; 2. Department of Endocrinology, School of Medicine, Mustafa Kemal University, Hatay, Turkey; 3. Department of Parasitology, School of Medicine, Mustafa Kemal University, Hatay, Turkey; 4. Department of Family Medicine, School of Medicine, Mustafa Kemal University, Hatay, Turkey

**Keywords:** *Demodex folliculorum*, Diabetes mellitus, Allergic rhinitis, Obesity

## Abstract

**Background:**

*Demodex* mites are permanent ectoparasites of human pilosebaceous unit. They mainly infect skin of the face and scalp. Many studies have shown higher density of the ectoparasites in diseased inflammatory skin than in normal skin. The aim of this study was to determine the frequency of *Demodex folliculorum* (DF) in treatment-resistant patients with the combination of allergic rhinitis (AR) and diabetes mellitus (DM).

**Method:**

This study was conducted in 2014–2017. It included 92 patients aged 18–70 years who presented at the Ear, Nose and Throat (ENT) and Endocrinology Polyclinics of Mustafa Kemal University Medical Faculty Hospital, Turkey. An age and gender matched control group was formed of 30 healthy individuals. To determine the presence of DF, a few eyelashes were taken from eyelids in both groups. Then samples were examined under a light microscope.

**Results:**

DF positivity was determined in 44 (47.8%) of the 92 patients and in 1 (3.3%) of the 30 control group subjects. In the patient group, DF positivity was evaluated as present in 14 (43.7%) of the DM patients, in 12 (40%) of the AR patients and in 18 (60%) of the AR+DM patients. Statistically significant DF incidence was found in all three patient groups compared to the control group (*P*=0.001). The incidence in AR + DM group was not different from other patient groups.

**Conclusion:**

DM, AR, advanced age and obesity had prepared the environment for *Demodex* infestations. This issue should be considered especially in treatment of patients with AR+DM.

## Introduction

Allergic rhinitis (AR) is a chronic disease characterised by itching of the nose, sneezing, discharge, obstruction and itching and watering eyes. It is estimated that 400 million people suffer from allergic rhinitis across the world, which affect approximately 20%–40%of the global population (1). As an individual's work and social life can be negatively affected, diagnosis and treatment demand attention. Diagnosis of AR is made from the anamnesis and physical examination. Characteristics such as family history, the presence of atopy, the age at the onset of symptoms and seasonal changes should be evaluated. In approximately 60%–70% of patients, there are accompanying ocular symptoms such as itching, redness and watering of the eyes (2). In the physical examination, diagnosis is made from the presence of purplishcoloured nasal mucosa, a pale appearance and serous discharge.

Diabetes mellitus (DM)is a disease which develops as a result of dysfunction in the activation or expression of insulin and in which impairments are seen in cellular and humoral immunity in the long term. Impairments in the chemotactic functions of leukocytes with polymorphous nucleus in DM patients increase the tendency for infection/infestations and may cause the development of complications (3).

Although the *Demodex* mite is present in most people, clinical findings emerge in a very small minority. Weakness occurring in the immune system causes *Demodex* proliferation and the passage to the dermis of the mite (4). It is thought that the disease is caused by a combination of factors such as mites in the dermis layer causing a mechanical obstruction in follicles, the forming of a foreign body reaction, the development of delayed type hypersensitivity against mite antigens and the expression of products causing local damage and bacterial proliferation (5).

In previous studies, DF has been seen to accompany skin diseases such as rosacea, blepharitis, perioral dermatitis, seborrheic dermatitis and acne vulgaris, and it has been shown that DF could contribute to the development of these diseases (5–7). The presence of DF has been shown at a high prevalence in immunosuppressive diseases such as chronic renal failure, DM and HIV (7).

DF is generally asymptomatic in healthy individuals. When the host has a weakened immune system, smokes cigarettes or drinks alcohol, consumes a high amount of spicy food, is of advanced age, under stress or has allergic skin diseases that disrupt the integrity of the skin, it has been shown in various studies that the incidence of DF and pathogenicity are increased (3–7).

To the best of our knowledge, there has been no study in literature that has investigated the incidence of DF in patients with the combination of AR+DM and therefore the aim of this study was to determine the incidence of DF in patients with both AR and DM.

## Materials and Methods

This study was conducted in 2014–2017. We included 92 patients aged 18–70 years who presented at the Ear, Nose and Throat (ENT) and Endocrinology Polyclinics of Mustafa Kemal University Medical Faculty Hospital, Turkey. AR was diagnosed with result of history and examinations. Type 2 diabetes mellitus was diagnosed ADA criteria (8). An age and gender-matched control group was formed of 30 healthy individuals. The demographic data of all the participants were recorded.

The 92 patients comprised 30 diagnosed with AR, 30 diagnosed with DM and 32 with AR+DM. The control group comprised 30 healthy individuals.

Patients were excluded if they were aged <18 years, had any dermatological disease on the body, and especially of the face, such as systemic lupus erythematosus, impetigo, acne vulgaris, perioral dermatitis, herpes infection or seborrheic dermatitis, if they were pregnant or breastfeeding, had any malignancy, smoked or drank alcohol, had any systemic disease such as chronic liver or kidney disease, were receiving chemotherapy or radiotherapy, were using topical anti-parasitic medication or had used topical or systemic antibiotics within the previous 6 weeks.

**Fig. 1:**
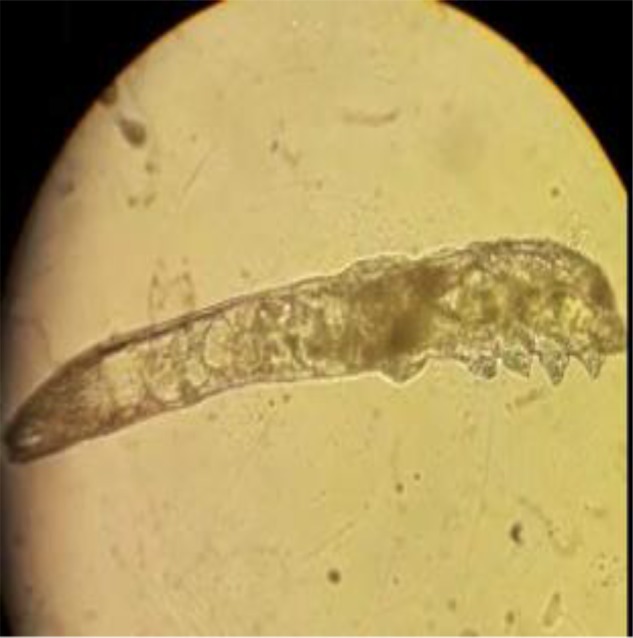
Light microscopic image of DF adult form (Original magnification 40×)

To determine the presence of DF, a few eyelashes from each eye are epilated and placed on the slide in glycerine liquid (9) and the samples were examined under a light microscope at ×40 and ×100 magnification. All samples were promptly evaluated for parasites using light microscopy (Fig. 1).

Approval for the study was granted by the Ethics Committe of Mustafa Kemal University Medical Faculty (2014/138). Informed consent was obtained from all participants. The study was conducted in accordance with the regulations of the Republic of Turkey and the principles of the Helsinki Declaration.

Analyses of the study data were made using SPSS for Windows vn. 21.0 (IBM, SPSS, Chicago, IL, USA). Continuous variables were stated as mean ± standard deviation (SD), and categorical variables as number (n) and percentage (%). Analysis of continuous variables between the groups was made using One-Way Variance Analysis (ANOVA) and for categorical variables, the Chi-Square test was applied. A value of *P*<0.05 was accepted as statistically significant.

## Results

The demographic and clinical characteristics of all the patients are shown in Table 1. The demographic and clinical characteristics of the patients determined with DF positivity are shown in Table 2. DF positivity was determined in 44 (47.8%) of the 92 patients and in 1 (3.3%) of the 30 control group subjects. The AR patients were observed to be younger and body mass index (BMI) values were lower. Both of them are statistically significant (respectively *P*=0.001, *P*=0.001), but no difference was determined between the groups in respect of age and BMI. In the patient group, DF positivity was evaluated as present in 14 (43.7%) of the DM patients, in 12 (40%) of the AR patients and in 18 (60%) of the AR+DM patients. When the control group is excluded from the analysis, In terms of demodex status, no statistically significant difference was found between patient groups (*P*=0.255). In smoking status, there is a significant difference in the DM + AR group among the other patient groups (*P*<0.05).

**Table 1:** Demographics features of the participants

***Variable***	***DM (n=32)***	***AR (n=30)***	***DM + AR (n=30)***	***Controls (n=30)***	***P-values***
Age (yr)	51.2 ± 12.4	29.2 ± 10.8	50.8 ± 11.6	40.9 ± 4.3	0.001^a^
Gender, F/M (n(%))	13 (40.6) / 19 (59.4)	15 (50) / 15 (50)	13 (43.3) / 17 (56.7)	14 (46.7) / 16 (53.3)	0.892
BMI (kg/m^2^)	30.1 ± 6.2	22.7 ± 3.3	32.2 ± 7.8	26.4 ± 4.5	0.001^a^
D. *folliculorum* status n (%)	14 (43.7)	12 (40)	18 (60)	1 (3.3)	0.001^b^

Data presented as mean ± SD or count (%).

a ANOVA,

b Chi-square

**Table 2:** Demographic and clinical features of *Demodex folliculorum* positive patients

***Variable***	***DM (n=14)***	***AR (n=12)***	***DM + AR (n=18)***	***P-values***
Gender M/F	8/6	7/5	9/9	0.879^b^
Age (yr)	51.4 ± 10.6	30.5 ± 13.8	51.3 ± 10.7	0.001^a^
BMI (kg/m^2^)	30.8 ± 6.0	23.0 ± 2.4	33.6 ± 8.3	0.001^a^
Smoking status (Yes/No)	2/12	1/11	4/14	0.583^b^
Alcohol status (Yes/No)	1/13	0/12	0/18	0.334^b^

Data presented as mean ± SD or count (%).

a ANOVA,

b Chi-square

## Discussion

In eyelash samples taken from patients with blepharitis and blepharoconjunctivitis were determined the incidence of *Demodex* as 29.7% and 9.0% respectively (10).Yengil et al determined a high incidence of DF in patients with AR (11). They reported DF positivity of 38.1% in the facial area, 50.8% in the eyelashes and 12.3% in the control group.In the current study, DF was observed at 40% in the AR patient group, which was consistent with the findings of this literature (11).

The incidence of DF was high in DM patients (12). In a study that evaluated 42 patients with type 2 DM, DF was observed at a higher rate in the cases with diabetes (54.8%) (13). In a study of patients with gestational diabetes, the prevalence of DF was statistically significantly higher in the patients with gestational diabetes than in the normal pregnancies (24.2% vs 3.3%) (14). In our study, DF positivity was determined as 43.7% in DM patients. Additionally, we obtained 60% DF pozitivity in patients with the combination of AR+DM. Impaired leukocyte-endothelial cell interactions and decreased quantity of leukocytes in inflammatory lesions have been identified in patients with diabetes. Moreover, chemotactic activity of neutrophils, function of mastocytes, release of cytokines (such as tumor necrosis factor alpha, interleukins and prostaglandins etc) were reduced in this groups. According to these results, it can be said that the impairments occurring in the immune system in patients with diabetes increase the susceptibility to infection and infestations (15–17).

In a study of patients with end stage chronic kidney disease (CKD), Demodex spp was found at 44.4% in the skin surface (18). In another study revealed that, DF positivity was determined at 51% in 80 patients diagnosed with rosacea, at 28% in 40 patients with eczema and at 31% in 40 patients with systemic lupus erythematosus (SLE) (19). Based on these results, *Demodex* incidence is increased particularly in diseases that weaken the immune system such as DM, CKD, SLE and in chronic skin diseases.

In our study, any correlation was not determined between DF positivity and gender. Similarly, Yamashita et al (13) found no difference in DF incidence between patient with diabetes and a healthy control group in respect of gender. In another study, significant difference was found between demodex frequency and gender (20). AR+DM group and the DM group where DF positivity was determined to be high, the age and BMI values of the patients were higher than those of the patients in the AR only group. Inceboz et al reported that *Demodex* incidence was higher in individual aged >45 years (21). The results of the current study are similar to previous findings in literature and support the view that DF positivity is seen more often in the advanced age and obese individuals.

There were some limitations to this study. Small-scale and it does not show any relationship with glycemic parameters (e.g. HbA1c, fasting plasma glucose) in patients with diabetes.

## Conclusion

*Demodex* should be investigated in patients with AR resistant to treatment. Especially in this group of advanced age and obesity.In patients with AR+DM combination, it should be taken into consideration that the high prevalence of *Demodex* infestation may exacerbate existing AR symptoms. If precautions are taken with the necessary anti-parasite treatment methods, there could be a positive improvement in the quality of life of this patient group.
